# Prevalence and correlates of severe problematic cannabis use: analysis of a population-based survey in Jamaica

**DOI:** 10.3389/fpsyt.2024.1465963

**Published:** 2024-11-12

**Authors:** Kunal Lalwani, Winston De La Haye, Kevon Kerr, Wendel Abel, Clayton Sewell

**Affiliations:** Department of Community Health and Psychiatry, The University of the West Indies, Mona, Jamaica

**Keywords:** problematic, cannabis, CAST, prevalence, correlates, population, Jamaica

## Abstract

**Background:**

There is an increasing demand for the treatment of problematic cannabis use (PCU) in low-income and middle-income countries. Jamaica’s historical inclination towards cannabis use underscores the need for research in addressing this issue.

**Objectives:**

To determine the prevalence and patterns of cannabis use and assess the sociodemographic factors, psychosocial correlates, knowledge and perceptions associated with severe PCU among Jamaicans using nationally representative data.

**Methods:**

This study involved a secondary data analysis of the last Jamaica National Drug Prevalence Survey. It included 786 participants who used cannabis in the past year and completed the Cannabis Abuse Screening Test (CAST). The CAST has been validated against the Diagnostic and Statistical Manual of Mental Disorders (DSM), with a score ≥ 7 meeting the criteria for severe PCU. CAST scores were dichotomized utilizing these thresholds, and data generated were analyzed with SPSS version 25 using Pearson’s χ2 test and logistic regression.

**Results:**

In the past year, 53.3% of Jamaicans who smoked cannabis had a score of 7 or higher on the CAST and smoked an average of 62.21 joints per month. Male respondents were twice as likely to have severe PCU as females. Additionally, young, middle, and older adults were respectively 3, 5 and 3 times more likely to report severe PCU compared to adolescent respondents. Participants who started cannabis use at 11 years and under, 12-17 years, and 18-25 years were respectively 5, 7 and 7 times more likely to report severe PCU than those at 26 years and older. Moreover, easy access to cannabis, a high perceived need for treatment, belief in increased national drug use prevalence, and awareness of the National Drug Control and Prevention Agency were associated with increased odds of reporting severe PCU.

**Conclusion:**

One out of every two Jamaicans who used cannabis in the past year reported severe PCU and smoked an average of two cannabis joints per day. Early initiation increases the risk of severe PCU. Accordingly, a public health approach involving multiple sectors is needed to provide treatment options.

## Introduction

The latest World Drug Report unequivocally ranks cannabis as the leading cause of substance use disorders in almost half of all surveyed countries (1). Due to the high prevalence of use, individuals seek attention for a wide range of adverse medical and mental health outcomes (2–4), intensifying a public health concern that continues to fuel the rising demand for treatment globally (5). While several individuals reportedly self-medicate with cannabis (6), evidentiary support as a therapeutic is unclear (7) and may indeed promote an elevated risk of suicidal ideations (8). Moreover, persistent cannabis use is associated with poor educational and social outcomes (2, 9), rising numbers of legal risks, vehicular accidents (10) and addiction (2, 11).

Jamaica has a significant history of cannabis use, chronicled by inextricably interwoven socio-cultural, economic and political processes (12). Contemporary scholarly publications examining cannabis use among the general population have provided critical insights. The most recent national survey indicated that 40% to 60% of adolescents and 60% to 80% of adults found it easy to access cannabis (13). Additionally, close to 30% of Jamaicans reported cannabis use at least once in their lives, with two out of three individuals progressing to chronic cannabis use (14). Moreover, greater than 40% of Jamaican drivers who currently smoke cannabis, admitted to driving under its influence, with over 85% engaging in heavy use (15). Cannabis was found to be the most commonly used drug among Jamaican polysubstance users (14). Reduced perceived risk of use, easy access, and early initiation of cannabis use in childhood and adolescence were identified as significant predictors of this harmful polysubstance habit (16). Amidst the current national cannabis policy, possession of small quantities (two ounces) for personal and medicinal use is decriminalized and permitted in Jamaica (17). Further investigation regarding the frequency, social contexts, knowledge and perceptions of cannabis use is likely to prove pivotal in predicting problematic cannabis use (PCU) within the Jamaican population.

To date, a few international studies have sought to highlight the beliefs and attitudes associated with PCU – defined as use that leads to negative health or social consequences (18–20). The lack of perceived need for treatment is consistently a significant barrier to seeking treatment for substance use disorders (21, 22). This becomes even more pertinent in the context of evolving cannabis policies, where shifting attitudes towards the risks and availability of cannabis could impact perceptions of needing treatment for PCU (23, 24). Indeed, recent research supports this point in reporting a higher likelihood of PCU among individuals who self-medicated with cannabis for physical, mental and sleep health reasons (25). Regarding sociodemographic and socioeconomic factors, prior research highlights an increased likelihood of PCU among males, younger populations, individuals with lower levels of education, unemployed persons and households with lower earned income (26–29). While recent data indicates an early age of onset and more frequent cannabis use as being correlated with PCU (30, 31), these individuals are more likely to make light of the associated harms (29, 32). Notwithstanding, most of these studies have utilized specific and smaller-sized samples, with no known literature derived from a general population within the Caribbean.

To address this gap, this study examined nationally representative data to achieve the following objectives: (1) determine the prevalence of cannabis use and PCU among Jamaicans; (2) examine the quantity of cannabis use among individuals with severe PCU; and (3) investigate the sociodemographic factors, psychosocial correlates, and respondent knowledge and perceptions associated with severe PCU. The data gathered is expected to reveal distinct profiles of problematic patterns of cannabis use behaviors among the Jamaican populace, which can inform targeted interventions and policies, especially in light of its strong national socio-cultural acceptance (12).

## Methodology

### Study setting, design and data source

**Figure 1:**
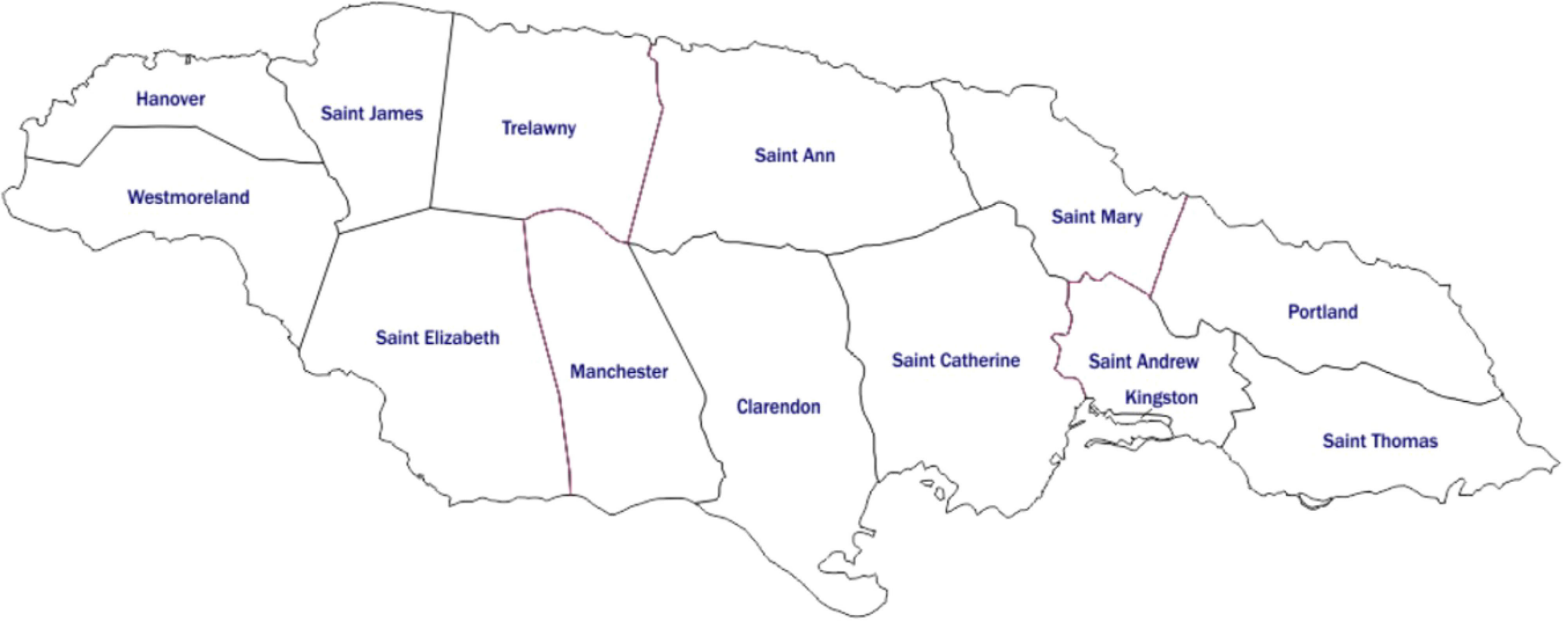
Map of Jamaica and parishes.

Jamaica is the third largest island in the West Indies, residence to just under three million citizens (33). The original study was the most recent National Drug Prevalence Survey (household survey) examining substance use patterns, for which the study design and data collection procedures have been previously illustrated (13). In summary, between April and July 2016, a nationally representative, interviewer-administered, cross-sectional survey was conducted to collect data from 4623 individuals between the ages of 12 and 65 years. Participants were enlisted using a multi-stage cluster sampling design based on a random selection of clusters or enumeration districts corresponding to the population’s size within each of Jamaica’s 14 parishes (**Figure 1**). Qualified interviewers employed structured questionnaires to obtain the requisite data. Sampling weights were determined and applied to account for selection probability, non-response, and population distribution, thus ensuring the accuracy of the findings (13). This study presents a secondary analysis of data extracted from the 2016 survey. The target population comprised 786 participants who used cannabis in the past year. This research selected variables relevant to cannabis use patterns, problematic cannabis use, age of first use, risk perception, perceived need for treatment, ease of access, beliefs and associated sociodemographic factors and psychosocial correlates. This study contained no identifying data on respondents, and there was no direct or indirect contact with any of the respondents.

### Study variables

This study used standardized items and operationalized measures to investigate the objectives. The dependent variable in the analyses was whether participants scored below or above a cut-off score of 7 on the full version of the Cannabis Abuse Screening Test (CAST) (34, 35). The independent variables examined included sociodemographic factors, psychosocial correlates, and respondent knowledge and perceptions, described below.

#### Cannabis use prevalence

The prevalence of cannabis use in the population was computed using the target variables for three different periods - lifetime (ever use), past year, and past month use. A response of “1” indicated “yes”, while “2” indicated “no”. In addition, respondents who reported cannabis use in the past year were asked, “when you do smoke cannabis, how many joints (spliffs) do you smoke a month?” to calculate the average quantity smoked per month.

#### Problematic cannabis use

The primary aim of CAST is to detect patterns of use that may lead to negative social or health outcomes, helping to identify individuals who may need further assessment or intervention. Designed using key criteria of the Diagnostic and Statistical Manual of Mental Disorders (DSM) (36), the questionnaire comprises a 6-item questionnaire that screens for problematic cannabis use in the general population by assessing the frequency of the following events within the past 12 months: (1) “have you smoked cannabis before midday?”; (2) “have you smoked cannabis when you were alone?”; (3) “have you had memory problems when you smoke cannabis?”; (4) “have friends or family members told you that you should reduce or stop your cannabis consumption?”; (5) “have you tried to reduce or stop your cannabis use without succeeding?” and (6) “have you had problems because of your cannabis use (argument, fight, accident, poor results at school, etc.)?”. The items are scored on a 5-point Likert scale from 0 (never) to 4 (very often), yielding a total ranging from 0 to 24. Several studies have validated the use of CAST to identify PCU in the general population by comparing it to the DSM criteria (34, 37). Additionally, studies have suggested that scores equal to or greater than 7 detect severe risk (34, 35, 38). In this study, CAST scores were dichotomized using these thresholds to identify individuals who meet the criteria for severe PCU among those participants who reported cannabis use in the past year.

#### Sociodemographic factors

Sociodemographic characteristics were included as covariates. Respondents were asked to state their sex (1=male and 0=female) and geographical location [recategorized into 1=urban, to represent urban parishes, and 0=rural, to represent rural parishes as previously reported (13)]. Respondents’ age was categorized into four groups representing adolescence (12-17 years), young adults (18-34 years), middle adults (35-54 years), and older adults (55-65 years) as established elsewhere (39, 40). Participants were asked “Are you the head of household”. The response options were 1=yes and 0=no. Educational status was assessed by asking respondents, “What is the highest educational level that you have achieved?” Ten response options were recategorized into 1=tertiary level and 0=below tertiary level. The item corresponding to marital status had seven response options recategorized into two choices: 1=married and 0=single. The item on religious affiliation had 26 response options that were recategorized as 2=Rastafarian, 1=other, and 0=not known. Employment status was re-categorized as 0=not employed, to include unemployed, students and retirees, and 1=employed as previously examined in the literature (41).

#### Psychosocial correlates

For this analysis, the psychosocial factors included age of initiation, friend and family drug use, cannabis accessibility and medicinal cannabis use. Participants reported their age at first cannabis use, which was categorized into four age groups: 11 years and under, 12-17 years, 18-25 years, and 26 years and older to demonstrate distinct phases of childhood, adolescence and adulthood (39). Regarding friend and family substance use habits, participants were asked if their friends or family members take illegal drugs such as cannabis and cocaine. The response options were limited to 1=yes and 0=no. As it pertains to medicinal use, respondents indicated 1=yes and 0=no to the question, “have you ever used cannabis for a medical condition?” Participants also rated ease of access to cannabis from the options: 1=easy, 2=difficult, 3=could not have access to and 4=don’t know. For the bivariate and multivariate analyses, the response options were recategorized into 1=easy and 0=not easy. The option “don’t know” was excluded as an underrepresented category with insufficient frequencies that may introduce variability in the interpretation of the results (42).

#### Knowledge and perceptions

The study assessed the perceived risks of using cannabis frequently and for medicine by asking respondents, “in your opinion, please indicate the risk level of smoking cannabis sometimes and often” and “in your opinion, please indicate the risk level of using cannabis for medicine sometimes and often”. Participants rated their risk level along a Likert scale from “no risk” to “high risk”. For the multivariate analysis, the responses were recategorized into three groups: 0=no risk, 1=low risk and 2=moderate to high-risk to examine respondents who had indicated some level of perceived risk. The “I don’t know the risk” option was excluded due to insufficient frequencies that may introduce bias in interpreting the results (42). In considering cannabis potency, participants were asked their opinion regarding the potency (strength) of the cannabis they used most recently. Six options were recategorized into 0=don’t know, 1=not strong, and 2=strong. Respondents who reported that there was never a time in the past 12 months when they felt they might need help or treatment for drug use were categorized as having “low perceived need”, as established elsewhere (43). An affirmative response was categorized as having a “high perceived need”. To investigate perceptions about the extent of the drug problem and prevalence of drug use in the country, individuals were asked “do you believe that, over the past few years, taking drugs in the country has increased, remained the same or declined?” and “do you believe that, in the coming years, the drug problem is going to get worse, remain the same or decline?”. The response “don’t know” was offered as a fourth option. For the multivariate analysis, the “don’t know” response was excluded to dually guard against variability in the interpretation of the results and demonstrate the association among respondents who had indicated some level of perception (42). Participants were asked to indicate whether they knew the National Council on Drug Abuse to demonstrate their awareness of the existence of Jamaica’s national drug control and prevention agency. Respondents were asked to indicate their level of awareness to demonstrate their knowledge of the changes to the Dangerous Drugs Act (DDA 2015) as it pertains to cannabis. Responses were recategorized into 1=high awareness (being aware of all/most changes) and 0=low awareness (being aware of few/no changes), a measure previously utilized in the literature (44).

### Statistical analysis

All statistical analysis was performed using the initial survey package in SPSS V.25. Sampling weights were determined and applied to the data to account for selection probability, non-response, and population distribution, ensuring that the weighted sample matched the population distribution of age and sex groups (13). Therefore, the models generated in this study considered clustering, stratification and weighting in the sample. Descriptive statistics included computing frequencies and percentages for categorical variables, as well as determining the prevalence of cannabis use among the population. Means and standard deviations (SD) were reported for select variables. Bivariate analyses using Pearson’s χ2 test were employed to test the association between independent variables with categorical data and the dichotomized CAST dependent variable. Subsequently, logistic regression was performed to include the statistically significant variables derived from the Chi-square analysis in a multivariate analysis. Multicollinearity between the study variables was assessed using variance inflation factor (VIF) (with multicollinearity defined as VIF>2.5) (45). Outliers and influential cases were detected using Cook’s distance and standardized residuals (46), with residuals beyond the range of −3 and +3 considered as likely outliers (47, 48). Additionally, the Hosmer and Lemeshow statistic was used to test the goodness of fit of the regression model (49). Odds ratios (ORs) and confidence intervals (CIs) were recorded for the likelihood of participants with severe PCU while controlling for the other variables in the model. A p<0.05 was considered statistically significant. Benjamini-Hochberg false discovery rate (FDR) correction was applied to provide adjusted p values (50). The data were presented in the form of tables and text. A 5% or less missing data rate was considered acceptable and likely inconsequential (51).

### Ethical approval

The Ministry of National Security in Jamaica approved the National Drug Use Prevalence Survey (13). Participants gave informed consent to participate in the study before taking part. The secondary data analysis was approved by the University of the West Indies Ethics Committee, Mona (Ref: CREC-MN.8, 2021/2022).

## Results

### Prevalence and quantity of cannabis use

**Table 1** displays the prevalence of cannabis use in the total population (n=4623). Within the total population, 786 persons or 17% had used cannabis in the past year. Among those in the population who reported lifetime use of cannabis, 53.9% reported use in the past month, and 60.1% reported use in the past year. All 786 participants who reported cannabis use in the past year completed the CAST questionnaire, giving a response rate of 100%. Among these respondents, 53.3% were considered to have severe problematic cannabis use. The mean number of cannabis joints smoked among these individuals was 62.21 joints per month. The mean number of cannabis joints smoked among those individuals whose CAST score was less than or equal to 6 was 32.90 joints per month.

**Table 1:** Prevalence of cannabis use and problematic cannabis use among the total population.

Cannabis Use	Frequency	Percentage
Population lifetime use	1307	28.3%
Population past month use	704	53.9%
Population past year use	786	60.1%
Population past year use and CAST ≤ 6	367	46.7%
Population past year use and CAST ≥ 7	419	53.3%

### Participant demographics, beliefs and psychosocial characteristics

**Tables 2**–**4** illustrate the demographic profile of Jamaicans who reported cannabis use in the past year, inclusive of associated psychosocial correlates, knowledge and perceptions regarding their substance use. The mean age of respondents was 35.61 years (SD ± 13.020). Most individuals were male (80.4%), young adults (48.2%), single (75.4%), employed (68.6%), and the head of their household (71.9%). Approximately 73.2% of respondents indicated the age of first cannabis use occurred under the age of 17. Regarding access to cannabis, the majority of respondents, 749 (95.3%), reported that accessing cannabis was “easy”. Concerning friends and family drug use, most respondents had friends or family who take illegal drugs such as cannabis and cocaine (90.1%). Notably, 95.5% of respondents reported a low perceived need for drug treatment in the preceding 12 months. Missing data was reported for less than 0.5% of responses regarding the variables pertaining to education level, marital status, perceived extent of the drug problem and drug use prevalence, respectively. Missing data were reported for less than 3% of responses related to religious affiliation. All other variables demonstrated a 100% response rate.

**Table 2:** Descriptive sociodemographic factors of the study sample.

Variable	Frequency	Percentage
*Gender*		
Male	632	80.4
Female	154	19.6
*Age*		
12-17 years	32	04.1
18-34 years	379	48.2
35-54 years	292	37.2
55-65 years	83	10.6
*Education level*		
Tertiary level	102	13.0
Below tertiary level	681	87.0
*Religious affiliation*		
Rastafarian	48	06.1
Other	475	60.4
Not known	263	33.5
*Employment*		
Employed	539	68.6
Not employed	247	31.4
*Marital Status*		
Single	591	75.4
Married	193	24.6
*Geographical location*		
Urban	289	36.8
Rural	497	63.2
*Head of the household*		
Yes	565	71.9
No	221	28.1

**Table 3:** Descriptive psychosocial correlates of the study sample.

Variable	Frequency	Percentage
*Age of initiation of cannabis*		
11 years and under	70	09.2
12-17 years	489	64.0
18-25 years	180	23.6
26 years and older	25	03.2
*Accessibility to cannabis*		
Easy	749	95.3
Difficult	21	02.7
Could not have access to	05	00.6
Don’t know	11	01.4
*Cannabis for medical use*		
Yes	199	25.3
No	587	74.7
*Friends/family who take illegal drugs*		
Yes	708	90.1
No	78	09.9

**Table 4:** Descriptive knowledge and perceptions of the study sample.

Variable	Frequency	Percentage
*Perceived cannabis potency*		
Strong	526	66.9
Not strong	248	31.6
Don’t know	12	01.5
***Risk perception of cannabis use***		
*Smoking sometimes*		
No risk	220	28.0
Low risk	253	32.2
Moderate risk	141	17.9
High risk	166	21.1
Don’t know the risk	06	00.8
*Smoking often*		
No risk	132	16.8
Low risk	172	21.9
Moderate risk	160	20.3
High risk	319	40.6
Don’t know the risk	03	00.4
*Using for medicine sometimes*		
No risk	412	52.4
Low risk	228	29.0
Moderate risk	69	08.8
High risk	66	08.4
Don’t know the risk	11	01.4
*Using for medicine often*		
No risk	346	44.0
Low risk	199	25.3
Moderate risk	93	11.8
High risk	138	17.6
Don’t know the risk	10	01.3
*Perceived need for drug treatment*		
Yes	35	04.5
No	751	95.5
*Awareness of changes to the DDA*		
High awareness	178	22.6
Low awareness	608	77.4
*Aware of the existence of the NCDA*		
Yes	130	16.5
No	656	83.5
*Perceived extent of drug prevalence*		
Increased	560	71.3
Remained the same	116	14.8
Declined	65	08.3
Don’t know	44	05.6
*Perceived extent of the drug problem*		
It’s going to get worse	522	66.7
It’s going to remain the same	144	18.4
It’s going to decline	62	07.9
Don’t know	55	07.0

### Factors associated with severe PCU

**Tables 5**–**7** show bivariate analyses of select sociodemographic, psychosocial, knowledge and perception factors and severe PCU. Pearson’s χ2 analysis revealed statistically significant associations between twelve of the variables and severe PCU. Participants who were male, between the ages of 18-34 years, employed and the head of the household were significantly associated with a greater prevalence of severe PCU, respectively (p<0.001, p<0.001, p=0.024, and p=0.003). Statistically significant associations were noted between severe PCU and the age of first onset of cannabis use and medical cannabis use (p<0.001 and p=0.002). Additionally, respondents who stated that cannabis was easy to access were significantly associated with severe PCU (p=0.022). Similar trends were observed for respondent perceptions and knowledge, as risk perception of using cannabis for medicine often, perceived need for treatment, perceived extent of the national drug use prevalence and drug problem, and awareness of the existence of the National Council on Drug Abuse in Jamaica demonstrated statistically significant associations with severe PCU (p<0.001, p=0.004, p<0.001, p=0.019, and 0.024). Using the Benjamini-Hochberg procedure, adjusted p values remained significant for all variables.

**Table 5:** χ2 analysis of sociodemographic factors associated with severe problematic cannabis use.

Factors	CAST ≤ 6	CAST ≥ 7	χ2	p value	adjusted p value
*Gender*			17.304	<0.001***	0.005**
Male	272 (74.1%)	360 (85.9%)			
Female	95 (25.9%)	59 (14.1%)			
*Age*			16.992	<0.001***	0.005**
12-17 years	25 (06.8%)	07 (01.7%)			
18-34 years	185 (50.4%)	194 (46.3%)			
35-54 years	121 (33.0%)	171 (40.8%)			
55-65 years	36 (09.8%)	47 (11.2%)			
*Education level*			0.030	0.863	0.863
Tertiary level	320 (87.2%)	361 (86.8%)			
Below tertiary level	47 (12.8%)	55 (13.2%)			
*Religious affiliation*			2.733	0.255	0.340
Rastafarian	20 (05.4%)	28 (06.7%)			
Other	233 (63.5%)	242 (57.8%)			
Not known	114 (31.1%)	149 (35.6%)			
*Employment*			5.105	0.024*	0.048*
Employed	237 (64.6%)	302 (72.1%)			
Not employed	130 (35.4%)	117 (27.9%)			
*Marital status*			0.050	0.823	0.941
Single	278 (75.7%)	313 (75.1%)			
Married	89 (24.3%)	104 (24.9%)			
*Geographical location*			2.172	0.141	0.226
Urban	125 (34.1%)	164 (39.1%)			
Rural	242 (65.9%)	255 (60.9%)			
*Head of the household*			8.948	0.003**	0.008**
Yes	245 (66.8%)	320 (76.4%)			
No	122 (33.2%)	99 (23.6%)			

*significant at the p <0.05, **significant at the p= 0.01 level, ***significant at the p<0.001 level.

**Table 6:** χ2 analysis of psychosocial correlates associated with severe problematic cannabis use.

Factors	CAST ≤ 6	CAST ≥ 7	χ2	p value	adjusted p value
*Age of initiation of cannabis*			20.386	<0.001***	0.004**
11 years and under	25 (07.1%)	45 (10.9%)			
12-17 years	214 (60.8%)	275 (66.7%)			
18-25 years	92 (26.1%)	88 (21.4%)			
26 years and older	21 (06.0%)	04 (01.0%)			
*Accessibility to cannabis*			9.667	0.022*	0.029*
Easy	342 (93.2%)	407 (97.1%)			
Difficult	14 (03.8%)	07 (01.7%)			
Could not have access to	05 (01.4%)	00 (00.0%)			
Don’t know	06 (01.6%)	05 (01.2%)			
*Cannabis for medical use*			9.674	0.002**	0.004**
Yes	74 (20.2%)	125 (29.8%)			
No	293 (79.8%)	294 (70.2%)			
*Friends/family who take illegal drugs*			0.143	0.706	0.706
Yes	329 (89.6%)	379 (90.5%)			
No	38 (10.4%)	40 (09.5%)			

*significant at the p <0.05, **significant at the p= 0.01 level, ***significant at the p<0.001 level.

**Table 7:** χ2 analysis of knowledge and perceptions associated with severe problematic cannabis use.

Factors	CAST ≤ 6	CAST ≥ 7	χ2	p value	adjusted p value
*Perceived cannabis potency*			2.803	0.246	0.351
Strong	238 (64.9%)	288 (68.7%)			
Not strong	121 (33.0%)	127 (30.3%)			
Don’t know	08 (02.2%)	04 (01.0%)			
***Risk perception of cannabis use***					
*Smoking sometimes*			4.937	0.294	0.368
No risk	92 (25.1%)	128 (30.5%)			
Low risk	117 (31.9%)	136 (32.5%)			
Moderate risk	74 (20.2%)	67 (16.0%)			
High risk	82 (22.3%)	84 (20.0%)			
Don’t know the risk	02 (00.5%)	04 (01.0%)			
*Smoking often*			2.263	0.688	0.688
No risk	55 (15.0%)	77 (18.4%)			
Low risk	79 (21.5%)	93 (22.2%)			
Moderate risk	76 (20.7%)	84 (20.0%)			
High risk	156 (42.5%)	163 (38.9%)			
Don’t know the risk	01 (00.3%)	02 (00.5%)			
*Using for medicine sometimes*			8.258	0.083	0.138
No risk	178 (48.5%)	234 (55.8%)			
Low risk	121 (33.0%)	107 (25.5%)			
Moderate risk	36 (09.8%)	33 (07.9%)			
High risk	26 (07.1%)	40 (09.5%)			
Don’t know the risk	06 (01.6%)	05 (01.2%)			
*Using for medicine often*			22.343	<0.001***	0.007**
No risk	142 (38.7%)	204 (48.7%)			
Low risk	119 (32.4%)	80 (19.1%)			
Moderate risk	46 (12.5%)	47 (11.2%)			
High risk	54 (14.7%)	84 (20.0%)			
Don’t know the risk	06 (01.6%)	04 (01.0%)			
*Perceived need for drug treatment*			8.361	0.004**	0.013*
Yes	08 (02.2%)	27 (06.4%)			
No	359 (97.8%)	392 (93.6%)			
*Awareness of changes to the DDA*			0.283	0.595	0.661
High awareness	80 (21.8%)	98 (23.4%)			
Low awareness	287 (78.2%)	321 (76.6%)			
*Aware of the existence of the NCDA*			5.069	0.024*	0.048*
Yes	49 (13.4%)	81 (19.3%)			
No	318 (86.6%)	338 (80.7%)			
*Perceived extent of drug use prevalence*			17.560	<0.001***	0.007**
Increased	246 (67.2%)	314 (74.9%)			
Remained the same	49 (13.4%)	67 (16.0%)			
Declined	42 (11.5%)	23 (05.5%)			
Don’t know	29 (07.9%)	15 (03.6%)			
*Perceived extent of the drug problem*			9.929	0.019*	0.048*
It’s going to get worse	233 (63.8%)	289 (69.1%)			
It’s going to remain the same	62 (17.0%)	82 (19.6%)			
It’s going to decline	36 (09.9%)	26 (06.2%)			
Don’t know	34 (09.3%)	21 (05.0%)			

*significant at the p <0.05, **significant at the p= 0.01 level, ***significant at the p<0.001 level.

### Predictors of severe PCU

**Table 8** shows a logistic regression analysis performed on the factors identified as statistically significant in the bivariate analyses, to identify which were predictive risks or protective factors. The model containing all twelve variables was statistically significant and predicted the dependent variable better than the intercept-only model alone (χ2 (19)=101.206, p<0.001), suggesting that the model was able to differentiate between individuals who have and do not have severe PCU. Additionally, the standardized residuals were not less than −3 or greater than 3, and Cook’s distance, was 0.01 at the maximum. The variance inflation factor values were all less than 1.34, suggesting that the assumption of multicollinearity was not violated. Furthermore, the Hosmer and Lemeshow goodness-of-fit statistic test shows the p value at 0.511 (p>0.05) which highlights that the model adequately fits the data.

**Table 8:** Regression model of factors associated with severe problematic cannabis use.

Variables	Estimate	OR	95% C.I.		p value	adjusted p value
			Lower	Upper		
Sex (female)		1				
Sex (male)	0.765	2.15	1.37	3.38	<0.001***	0.013*
Age (12-17 years)		1				
Age (18-34 years)	1.298	3.66	1.32	10.17	0.013*	0.035*
Age (35-54 years)	1.664	5.28	1.81	15.44	0.002**	0.013*
Age (55-65 years)	1.234	3.44	1.08	10.97	0.037*	0.059
Employment (not employed)		1				
Employment (employed)	-0.063	0.94	0.63	1.41	0.761	0.761
Head of the household (no)		1				
Head of the household (yes)	0.156	1.17	0.77	1.77	0.459	0.335
Age of initiation of cannabis (26 years and older)		1				
Age of initiation of cannabis (18-25 years)	1.977	7.22	1.81	28.81	0.005**	0.016*
Age of initiation of cannabis (12-17 years)	1.989	7.31	2.01	26.63	0.003**	0.013*
Age of initiation of cannabis (11 years and under)	1.621	5.06	1.36	18.81	0.016*	0.034*
Accessibility of cannabis (not easy)		1				
Accessibility of cannabis (easy)	1.080	2.94	1.02	8.53	0.047*	0.069
Risk perception of using cannabis for medicine often (no risk)		1				
Risk perception of using cannabis for medicine often (low risk)	-0.735	0.48	0.32	0.73	<0.001***	0.013*
Risk perception of using cannabis for medicine often (moderate to high risk)	-0.160	0.85	0.58	1.26	0.424	0.537
Cannabis for medical use (no)		1				
Cannabis for medical use (yes)	0.270	1.31	0.88	1.96	0.189	0.257
Perceived need for drug treatment (no)		1				
Perceived need for drug treatment (yes)	0.995	2.71	1.14	6.42	0.024*	0.041*
Aware of the existence of the NCDA (no)		1				
Aware of the existence of the NCDA (yes)	0.569	1.77	1.12	2.78	0.014*	0.033*
Perceived extent of drug use prevalence (declined)		1				
Perceived extent of drug use prevalence (remained the same)	0.800	2.23	1.13	4.39	0.021*	0.040*
Perceived extent of drug use prevalence (increased)	1.141	3.13	1.47	6.69	0.003**	0.013*
Perceived extent of the drug problem (it’s going to decline)		1				
Perceived extent of the drug problem (it’s going to remain the same)	0.191	1.21	0.61	2.41	0.588	0.657
Perceived extent of the drug problem (it’s going to increase)	0.118	1.13	0.54	2.35	0.752	0.794

*significant at the p <0.05, **significant at the p=0.01 level, ***significant at the p<0.001 level.

Multivariate analysis indicated that male respondents who used cannabis in the past year were 2.15 times (95% CI 1.37 to 3.38, p<0.001) more likely than their female counterparts to report severe PCU. Young, middle and older adult respondents were 3.66 (95% CI 1.32 to 10.17, p=0.013), 5.28 (95% CI 1.81 to 15.44, p=0.002) and 3.44 (95% CI 1.08 to 10.97, p=0.037) times more likely to have severe PCU than those between 12-17 years of age. Respondents commencing cannabis use at 11 years and under, between 12 and 17, and 18 and 25 years were five to seven (OR 5.06, 95% CI 1.36 to 18.81, p=0.016; OR 7.31, 95% CI 2.01 to 26.63, p=0.003; and OR 7.22, 95% CI 1.81 to 28.81, p=0.005) times more likely to report severe PCU than participants initiating cannabis use 26 years and older. The model also highlighted that those respondents who indicated cannabis access as being easy were associated with 2.94 (95% CI 1.02 to 8.53, p=0.047) increased odds of reporting severe PCU than those who reported access as not easy. In addition, respondents who reported the perceived risk of using cannabis for medicine often as being harmful reduced the risk of severe PCU. Respondents who indicated a high perceived need for treatment (OR 2.71, 95% CI 1.14 to 6.42, p=0.024) and perceived escalation in the national drug use prevalence (OR 3.13, 95% CI 1.47 to 6.69, p=0003), were associated with increased odds of severe PCU. Those respondents who indicated that they were aware of the national drug control and prevention agency were 1.77 (95% CI 1.12 to 2.78, p=0.014) times more likely to report severe PCU than those who were unaware. Although, being employed reduced the risk of severe PCU among these respondents, this inverse association was not statistically significant. Using the Benjamini-Hochberg procedure, adjusted p values remained significant for all variables, except age group 55-65 years and accessibility of cannabis, which were approaching significance (0.059 and 0.069).

## Discussion

Globally, CAST has been used to estimate the prevalence of problematic cannabis use among adolescents, young adults and general populations. The prevalence among adolescents ranged from 1.4% to 7.3% across several European countries in the most recent European School Survey Project on Alcohol and Other Drugs study (52). In Norway, approximately 6% of young adults in a population survey had a CAST score equal to or greater than 7 to meet the criteria for severe PCU (53). In comparison to a general population survey in France, the prevalence of severe PCU among those who reported cannabis use in the past year in Jamaica is approximately ten times higher (5.5% and 53.5% respectively) (35). The high proclivity to use among Jamaicans is likely multi-factorial. Jamaica is well-known for its strong cultural connection to cannabis use and cultivation, particularly within the Rastafari faith and traditional folk medicine (12, 54). In 2015, the country became the first nation in the Caribbean to revise its Dangerous Drugs Act (DDA) to decriminalize personal possession and allow home cultivation for personal use (17). As a result, adults can legally possess up to 2 ounces of cannabis and grow up to five plants per household. The amendment also permitted Rastafarians to use cannabis for religious purposes without restrictions and established the Cannabis Licensing Authority to oversee the production and sale of cannabis products for medicinal use. Legal cannabis dispensaries have since been established across the island, where adults can purchase cannabis with a doctor’s recommendation (17). Accordingly, the changes made to the DDA are likely to have significantly contributed to normalizing the status of cannabis in Jamaica. To the best of the researchers’ knowledge, this study is the first to report the prevalence of severe PCU and assess its associated factors in Jamaica and the Caribbean region.

Of interest, individuals with severe PCU smoked an average of 2 cannabis joints daily. While factors such as dosage, delta-9-tetrahydrocannabinol (THC) potency and method of administration may influence standardized measures of cannabis consumption (55), limited research has considered the impact of quantity on harm outcomes. This study addresses this gap by fulfilling critical parameters in deriving a measure from respondents in a general population, using an internationally validated screening tool (CAST). In addition, the findings presented were accrued in a legal and socio-culturally tolerant LMIC setting and are likely to be of value in public health and research, considering that the latest iteration of the Global Burden of Disease indicates that the burden of problematic use is shifting towards developing nations (56).

Indeed, the high frequency of severe PCU among Jamaicans, especially men, represents a unique and worrying public health issue associated with substantial co-morbidity and disability (57, 58). Numerous drug research studies conducted both internationally (59–62) and in Jamaica (13, 14, 63, 64), have demonstrated a similar sex pattern, suggesting that societal norms and sex roles are likely significant factors. As such, the observed difference in this study can be invariably attributed to the higher tendency for risk-taking behavior seen in males (65), often viewed as an enhanced demonstration of masculinity, and the concomitant societal prejudice associated with female drug use (66, 67).

Focusing on other factors, this study highlighted two important and inter-related elements to developing severe PCU - age of initiation and current age. The current finding, that beginning cannabis use in childhood and adolescence is associated with developing problematic use in later life, is well-supported by earlier literature (68–71). More compelling, however, is that initiating use in early adulthood (18-25 years) was strongly predictive of developing severe PCU and alarmingly comparable to starting use in adolescence among the Jamaican population. While early use is associated with greater health consequences, poorer academic or work performance, and a higher probability of developing a polysubstance habit (2), cannabis use during early adulthood might have negative impacts on psychosocial well-being, increase disease risk in later adulthood, and complicate typical life goals such as raising children and achieving career success (72). It is notable, therefore, that Jamaican adults, particularly middle-aged ones, demonstrated a considerably higher prevalence of severe PCU as compared to adolescents. These findings succinctly indicate that associations exist not only for adolescents, but also for individuals who commence cannabis use during early adulthood, and underscore the importance of developing distinct national public education initiatives that cater to and provide information regarding the impact of cannabis use across two different but critical developmental life periods.

Pre-existing evidence suggests that cannabis law reform leads to increased use among the general population (73). In this study, respondents who indicated cannabis access as easy were more likely to report severe PCU. The present findings extend prior drug research in Jamaica in demonstrating the ease of access to cannabis as a driving cause of substance use (13) and abuse (16). Moreover, a number of studies report an earlier age of first use and elevated levels of problematic use in territories that enact laws that increase the avenues through which to acquire cannabis, whether by home cultivation or via established medical dispensaries (74–77). Ergo, the access mechanisms for cannabis influenced by decriminalization in Jamaica, to permit the cultivation of up to five cannabis plants for personal use and the formation of a legal medicinal cannabis industry, as similar contributors to this study’s reported levels of use and problematic use in the population, cannot be summarily dismissed. Undoubtedly, this advancement has made it even easier to access cannabis.

Public attitudes and beliefs toward the issue of drugs are critical to the success of drug-related policies, which are likely to prove uncertain without their support (78). As previous studies suggest, perception of need is a crucial factor in deciding whether to seek help and is a necessary step in the process of changing addictive behaviors (21, 22, 79). The low prevalence of perceived need for treatment is consistently a major barrier to seeking treatment for substance use disorders (21, 22). Respondents in this study who had severe PCU, however, were more likely to report a high perceived need for treatment. Moreover, these persons were also more likely to be aware of the country’s national drug control and prevention agency, the National Council on Drug Abuse (NCDA). This finding is particularly notable, as previous research has contrastingly indicated that more than 80% of Jamaica’s general population were unaware of the NCDA (13). These results highlight that many of the barriers to care that people with severe PCU face at this stage are likely structural and financial. While acute drug treatment services are available in private settings that may ultimately be cost prohibitive, the only hospital-based drug treatment facility is a 6-bed unit located at the University Hospital of the West Indies in Kingston, the capital city of Jamaica (80). The available services or lack thereof, serve as a deterrent to seeking treatment and make access to care a challenge, underscoring a need for the establishment of additional hospital-based drug treatment facilities in and around the island. Additionally, the present analysis denotes that individuals with severe PCU indicated a high level of concern about the current drug situation in reporting that they perceived the national prevalence of drug use had been progressively increasing. Intervention at this stage must utilize the opportunity to incorporate the perspectives and personal lived experiences of these individuals to gain valuable insights into current trends, and to effectively translate research findings into impactful public policies and service practices that are likely to be beneficial in addressing the drug’s use (81).

Legal cannabis reforms can have varying public health impacts due to increased accessibility (82). Cumulatively, the findings of this research represent an inaugural viewpoint that encourages a public health approach. The framework galvanizes a collaborative union of key perspectives from persons with lived experience, policymakers, physicians, public health professionals, social service providers, economists and educators in developing evidence-based policies, procedures, and programs to address PCU and improve population well-being (83, 84). In tandem, the findings encourage a further call to review the current national cannabis policy to include more robust regulatory mechanisms and disseminate a public education program on the associated risks as part of an overall prevention and harm reduction effort. The data gleaned may provide the groundwork in encouraging a cannabis policy review for other territories that have similarly endorsed decriminalization or legalization.

Despite the important contribution that this study makes, it is important to consider the findings in relation to their strengths and limitations. The study’s limitations include the use of self-report measures, which rely on participants’ memory and subjectivity and may introduce a recall bias. Furthermore, many of the survey participants may have been inclined to provide answers that were deemed “socially acceptable”, potentially introducing a response bias. It is important to note that this research was unable to establish causal relationships because the data collected was cross-sectional in nature. Additionally, only four of the nineteen variables studied were psychosocial correlates. Notwithstanding, one of the strengths of the study is that the validated CAST improved the reliability of the findings, as it is widely used in national population sample studies worldwide (85). The random selection of survey respondents in the initial study and the use of a large population sample for the current study were significant strengths, producing results that may be generalized to the national population. Although the data analyzed in this study were collected in 2016, the findings are immensely valuable in laying the foundation for further research to continually fill a knowledge gap, especially in low-income and middle-income countries where global research is limited.

## Conclusion

One out of every two Jamaicans who used cannabis in the past year reported severe problematic use. This study sheds light on these individuals’ drug-related knowledge and perceptions that may be of immense value in improving treatment options at a clinical level and in addressing the current national cannabis policy at a legislative level. Policy perspectives need to adopt a public health approach, integrate harm reduction strategies, and not solely focus on preventive measures to prevent escalating habits. This is especially important given the current legal and socio-cultural contexts of cannabis use within the Jamaican population. Finally, the average number of cannabis joints smoked daily among those with severe PCU was two. This measure of quantity may be an effective way to quantify cannabis consumption in further research examining cannabis-related problems. While more research to further validate and establish such a measure across different populations is essential, incorporating this information can play a vital role in developing accurate public health messages for the Jamaican populace and informing cannabis policy.

## Data Availability

The datasets presented in this article are not readily available because data were used under license for the current study, and so are not publicly available. Data are however available from the authors upon reasonable request and with permission of the National Council on Drug Abuse, Jamaica, and the Inter-American Drug Abuse Control Commission (CICAD). Requests to access the datasets should be directed to Mrs. Uki Atkinson, research analyst, at uatkinson@ncda.org.jm.
